# Serum copper-to-zinc-ratio and risk of incident infection in men: the Kuopio Ischaemic Heart Disease Risk Factor Study

**DOI:** 10.1007/s10654-020-00644-1

**Published:** 2020-05-13

**Authors:** Jaakko T. Laine, Tomi-Pekka Tuomainen, Jukka T. Salonen, Jyrki K. Virtanen

**Affiliations:** 1 Institute of Public Health and Clinical Nutrition, University of Eastern Finland, P.O. Box 1627, 70211 Kuopio, Finland; 2 Department of Public Health, Faculty of Medicine, University of Helsinki, Helsinki, Finland; 3 MAS-Metabolic Analytical Services Oy, Helsinki, Finland

**Keywords:** Copper, Zinc, Infections, Population study, Prospective study

## Abstract

Infections are one of the main causes of mortality in elderly due to the decrease of immune response, for which copper (Cu) and zinc (Zn) are claimed to be crucial. High serum copper-to-zinc-ratio **(**Cu/Zn-ratio) has been reported with infections, but little is known whether it could also predict the incidence of infections. The study cohort consisted of 1975 men aged 42–60 years and free of severe infectious disease at baseline in 1984–1989 from the prospective population-based Kuopio Ischaemic﻿ Heart Disease Risk Factor Study. The main outcome was an incident infection leading to hospitalization. Cox proportional hazards regression models were used for statistical analysis. During the average follow-up of 19.2 years, 636 incident first cases of infections were diagnosed. The hazard ratio (HR) of developing an incident infectious disease in the highest compared to the lowest Cu/Zn-ratio quartile after adjustment for age and baseline examination year was 1.35 [95% confidence interval (CI) = 1.07–1.69, *P*-trend across quartiles = 0.005]. The association was slightly attenuated after additional adjustment for potential confounders (HR = 1.21, 95% CI = 0.96–1.53, *P*-trend = 0.054). Furthermore, higher serum Cu concentration was associated with higher risk of an incident infection. The multivariable-adjusted HR was 1.39 (95% CI = 1.10–1.75, *P*-trend = 0.005) in the highest versus the lowest serum Cu quartile. Serum Zn concentration was not associated with the risk (multivariable-adjusted extreme-quartile HR = 0.83, 95% CI = 0.67–1.04, *P*-trend = 0.218). In conclusion, our data suggest that an increased Cu/Zn-ratio and especially serum Cu concentration are associated with increased risk of incident infections in middle-aged and older men in Eastern Finland.

**Electronic supplementary material:**

The online version of this article (10.1007/s10654-020-00644-1) contains supplementary material, which is available to authorized users.

## Introduction

Due to the rapidly increasing life expectancy in developed countries, the number of elderly people is rising [1]. Elderly are more susceptible to infections and more often prone to severe complications than the younger individuals [2]. In the population of 65 years and older, infections are responsible not only for one-third of deaths [3] but also for many inpatient stays [4]. One of the major factors for the higher susceptibility to infections is the age-related weakening of the immune system (immunosenescence) [5].

Copper (Cu) and zinc (Zn) are essential micronutrients and crucial components for the development and maintenance of the immune and antioxidative defense system. Cu and Zn have an impact especially on the cell-mediated immune reactions of both innate and acquired immune defense [6, 7]. Earlier studies have shown that deficiencies of Cu and Zn predispose to infections, but also systemic inflammation and infections induce reduction of serum Zn concentration during the acute phase response due to the redistribution of the serum Zn into the liver and other tissues [7]. Furthermore, acute infections lead to an increase of serum Cu concentration [8]. Both responses result in an increased serum Cu/Zn-ratio.

Apart from a clear deficiency or supplementation, the serum concentrations of Zn and Cu are not significantly affected by dietary intakes, but are rather a result of pathophysiological changes in the body [9, 10]. It has been proposed that serum Cu/Zn-ratio would be a superior prognostic and predictive marker for several pathological and pre-pathological stages, as it reflects the reciprocal reaction of Cu and Zn better than serum Cu or Zn concentrations alone [9]. High serum Cu/Zn-ratio is claimed to be associated with higher risk of cardiovascular mortality [11, 12], different cancers [13, 14], and all-cause mortality [15]. A higher plasma Cu/Zn-ratio was discovered among hospitalized people aged 70 or older than in their healthy controls [16]. Furthermore, it was found that Cu/Zn-ratio significantly increases with aging, as Zn concentration decreases and Cu concentration increases with advancing age [17]. A mild Zn deficiency has been claimed to be quite prevalent in the elderly population even in developed countries [5, 18]. Similarly, decreased serum Zn concentration and increased Cu/Zn-ratio has been observed due to a chronic exposure to cadmium especially in smokers [19].

Furthermore, little is known whether the serum Cu/Zn-ratio could have an impact on the risk of getting an infection. Therefore, we investigated the relation of serum Cu/Zn-ratio with the risk of incident infections in a long-term prospective cohort study in middle-aged and older Finnish men.

## Materials and methods

### Study population

The Kuopio ischaemic heart disease risk factor (KIHD) Study was designed to investigate risk factors for cardiovascular disease, atherosclerosis, and related outcomes in a population-based sample of men from eastern Finland [20]. The baseline examinations were carried out in 1984–1989. A total of 2682 men who were 42, 48, 54, or 60 years old at baseline (83% of those eligible) were recruited in 2 cohorts. The first cohort consisted of 1166 men who were 54 years old and enrolled in 1984–1986, and the second cohort included 1516 men who were 42, 48, 54, or 60 years old and enrolled in 1986–1989. The baseline characteristics of the entire study population were described previously [20].

Data on serum Cu and Zn were available for 2573 men. Participants who had the serum CRP > 10 mg/L (n = 68), or those with a diagnosis of chronic diseases that are associated with increased risk of infections, such as chronic bronchitis (n = 195), lung tuberculosis (n = 87), bronchial asthma (n = 61), liver or pancreas disease (n = 30) or kidney stones (n = 122), chronic prostatitis (n = 2), thyroid therapy (n = 17), or rheumatoid arthritis (n = 16) at entry, were excluded, leaving 1975 men for the analysis. In the sensitivity analyses, we further excluded men with potentially increased risk of infections, including men with history of ischaemic heart disease, stroke, cancer or diabetes (n = 551), leaving 1424 men for these analyses.

### Data collection

Fasting venous blood samples were drawn between 08.00 and 10.00 at baseline. Subjects were instructed to abstain from ingesting alcohol for 3 days and from smoking and eating for 12 h before providing the sample. Copper-free needles and tubes were used for collecting and storing the blood samples. The subjects rested in a supine position for 30 min before blood sampling. A tourniquet was not used.

Detailed descriptions of the assessment of medical history and medications [21], family history of diseases [21], smoking [21], alcohol consumption [21], serum ferritin [21], and physical activity [22], at baseline have been published. Education and annual income were obtained from a self-administered questionnaire. BMI was computed as the ratio of weight in kilograms to the square of height in meters. Dietary intakes at baseline were assessed with 4-day food records [23]. Serum Cu and Zn concentrations were determined by atomic absorption spectrometry from frozen samples stored at − 20 °C for 1–5 years prior to analyses. Serum zinc concentrations were determined in the same batches with copper, method for which has been described before [24]. The PerkinElmer 306 atomic absorption spectrophotometer (Norwalk, Connecticut, USA) was used for measurements (Seronorm Nycomed, Oslo, Norway). Control serum samples were included in all daily batches. The reference standards were dissolved in 5% glycerol. The between-batch coefficient of variation was 4.0%. An immunometric assay (Immulite High Sensitivity C-reactive Protein Assay; DPC, Los Angeles, USA) was used to measure serum high-sensitivity CRP.

### Ascertainment of follow-up events

Follow-up diagnoses until 31 December 2012 were collected by record linkage to the national hospital discharge register using the Finnish personal identification code. International Classification of Diseases (ICD)-8 codes 002, 003, 008, 009, 035, 038, 053, 057, 060, 070, 078, 079, 380, 381, 420, 421, 460, 461, 465, 466, 472, 473, 480, 482, 485, 486, 590, 595, 601, 604, 680–682 and ICD-10 codes A04, A07–A09, A32, A40, A41, A46, A49, A69, A98, B00, B02, B24, B25, B34, B99, G03, H60, I33, I40, J01, J06, J13, J15, J18, J20–J22, L02, L03, L30, M00, M01, N10, N30, N39, N41and N45 were used to indicate an infection. Only the first incident event diagnosis was counted.

### Statistical analysis

The distributions of the baseline characteristics in quartiles of serum Cu/Zn-ratio were presented as means [± standard deviations (SD)] or percentages. The associations between serum Cu/Zn-ratio and baseline characteristics were analysed by using Chi square tests (for categorical variables) and by linear regression (for continuous variables).

Multivariable-adjusted Cox proportional hazards regression models were used to estimate the hazard ratios in quartiles of baseline serum Cu/Zn-ratio and in quartiles of Cu and Zn concentrations. Participants contributed follow-up time until the first diagnosis of incident infectious disease, death or the end of follow-up, which ever came first. The confounders were selected on the basis of risk factors for infections. Model 1 included age (years) and baseline examination year. Model 2 included model 1 plus history of ischemic heart disease, stroke, cancer or diabetes (yes/no); smoking (never smoker, previous smoker, current smoker < 20 cigarettes/day, current smoker ≥ 20 cigarettes/day); education (years); income (euros/year); intake of alcohol (g/week); leisure-time physical activity (kcal/day); and BMI (kg/m^2^). The cohort mean was used to replace missing values (< 2.3%). Potential nonlinear associations were assessed semiparametrically using restricted cubic splines. Statistical significance of the interactions with serum albumin and BMI medians and with smoking status (current smokers vs. others) on a multiplicative scale was assessed by likelihood ratio tests using a cross-product term with Cu/Zn-ratio and Cu and Zn concentrations as continuous variables. Similar analyses were conducted by stratifying the analyses with serum Cu concentration by the median of serum Zn concentration and vice versa. Statistical analyses were performed using SPSS software version 25 for Macintosh (Armonk, NY, IBM Corp.) and Stata 14.1 (Stata Corp., College Station, TX; for spline analysis). All *P*-values were two-sided.

## Results

The mean age of the 1975 men was 52.8 years and the mean serum Cu/Zn-ratio was 1.2 (SD 0.2, range 0.6–3.0). The mean serum Cu and Zn concentrations were 17.4 μmol/L (SD 2.7 μmol/L, range 7.2–36.5 μmol/L) and 14.4 μmol/L (SD 1.8 μmol/L, range 8.3–24.8 μmol/L), respectively. The baseline characteristics of the cohort members according to the quartiles of serum Cu/Zn-ratio are shown in Table 1. Men with a higher serum Cu/Zn-ratio were more likely to be older, be current smokers, consume more alcohol, be less educated and have a lower income than men with a lower serum Cu/Zn-ratio. Additionally, men with a higher Cu/Zn-ratio were more likely to have a higher serum C-reactive protein concentration and a lower serum albumin concentration. Among dietary factors, men with a higher serum Cu/Zn-ratio had higher intake of fish and butter and lower intakes of vegetable margarines and oils, grains and fruits, berries, vegetables and roots. There was no appreciable difference in Zn intake between the groups. We did not have information on Cu intake.

**Table 1:** Baseline characteristics of 1975 KIHD participants by quartiles of serum Cu/Zn-ratio

Characteristic	Serum Cu/Zn-ratio quartile				*P*-trend^a^
	1 (0.59–1.03)	2 (1.04–1.17)	3 (1.18–1.33)	4 (1.34–2.97)	
Participants, n	494	493	495	493	
Age, years	52.0 (5.5)^b^	52.6 (5.2)	52.8 (5.1)	53.7 (5.0)	< 0.001
Body mass index, kg/m^2^	26.7 (3.3)	27.0 (3.7)	26.8 (3.4)	26.8 (3.7)	0.990
Leisure time physical activity, kcal/day	135 (151)	155 (177)	139 (171)	128 (170)	0.235
Education, years	9.4 (4.0)	8.7 (3.5)	8.6 (3.3)	8.2 (3.1)	< 0.001
Income, Euros/year	14 745 (9937)	13 933 (9514)	13 427 (7910)	12 300 (9004)	< 0.001
Alcohol intake, g/week	56 (103)	57 (86)	72 (107)	108 (157)	< 0.001
Current smoker, %	21	24	30	41	< 0.001
Multivitamin use, %	2.0	1.0	0.2	1.0	0.049
History of ischemic heart disease, stroke, cancer or diabetes, %	24	28	28	33	0.003
Serum C-reactive protein, mg/L	1.2 (1.1)	1.5 (1.4)	1.8 (1.6)	2.7 (2.3)	< 0.001
Serum ferritin, nmol/L	0.4 (0.3)	0.4 (0.3)	0.4 (0.4)	0.4 (0.3)	0.242
Serum albumin, g/L	43.2 (3.4)	42.8 (3.5)	42.1 (3.6)	41.6 (3.5)	< 0.001
Dietary intakes					
Energy intake, kcal/day	2455 (588)	2486 (585)	2423 (620)	2405 (656)	0.083
Meat g/d^c^	160 (88)	158 (76)	157 (80)	161 (76)	0.916
Dairy g/d	709 (362)	723 (350)	711 (361)	702 (367)	0.660
Fish g/d	40 (48)	44 (52)	46 (51)	52 (59)	< 0.001
Eggs, g/d	34 (26)	33 (26)	32 (24)	31 (26)	0.074
Grains, g/d	262 (93)	266 (93)	252 (92)	241 (95)	< 0.001
Vegetable margarines and oils, g/d	22 (18)	20 (17)	19 (16)	17 (15)	< 0.001
Butter, g/d	29 (25)	34 (27)	32 (25)	36 (29)	0.002
Fruits, berries, vegetables, roots, g/d	437 (175)	433 (174)	418 (193)	386 (165)	< 0.001
Zinc, mg/d^d^	15 (4)	15 (3)	15 (3)	15 (3)	0.302

^a^*P*-trend was calculated by using linear regression (continuous variables) or by Chi square test (categorical variables)

^b^Values are means (SD) or percentages

^c^Includes red meat, white meat, game and offal

^d^Values adjusted for total energy intake using the residual method

**Fig. 1:**
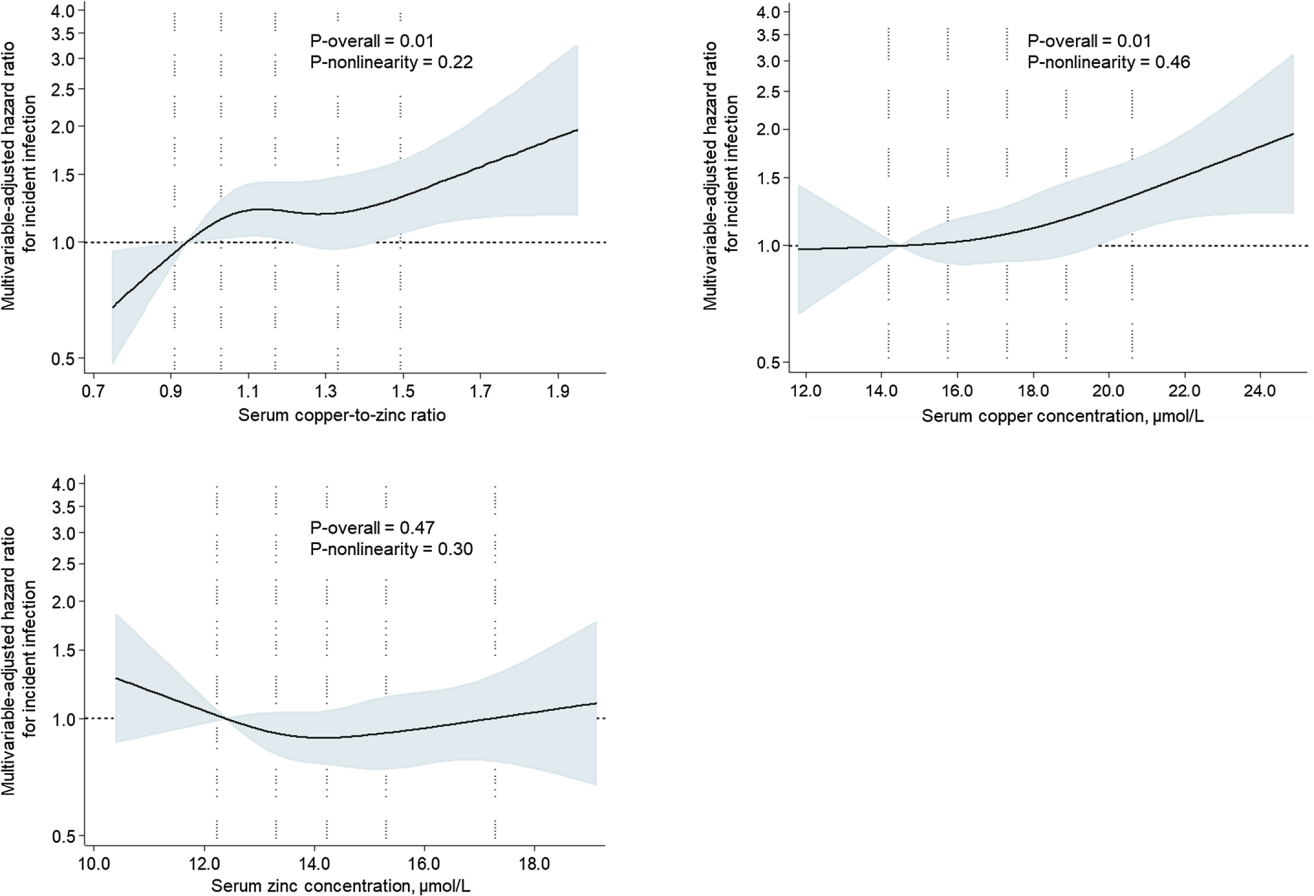
Hazard ratios of the serum copper-to-zinc-ratio and serum copper and serum zinc with risk of an incident infection among 1975 men, evaluated by restricted cubic splines from Cox proportional hazards models. The model is adjusted for age (years), examination year, history of coronary heart disease, stroke, cancer or diabetes (yes/no); smoking (never smoker, previous smoker, current smoker < 20 cigarettes/day, current smoker > 20 cigarettes/day); education (years); income (euros/year); intake of alcohol (g/week); leisure-time physical activity (kcal/day); and body mass index. The solid line represents the central risk estimate and the shaded area the 95% confidence interval. The dotted vertical lines correspond to 10th, 25th, 50th, 75th and 90th percentile of the serum copper-to-zinc-ratio or serum copper or zinc concentrations

During the average follow-up time of 19.2 years (SD: 8.0 years; minimum–maximum 0.1–28.7 years), 636 first infections requiring hospitalization occurred. Of these, 171 (26.9%) were pneumonia, 81 (12.7%) were erysipelas and 34 (5.3%) were acute bronchitis. After adjustment for age and baseline examination year, those in the highest versus the lowest Cu/Zn-ratio had 35% higher risk of an incident infection (95% CI = 7–69%; *P*-trend across quartiles 0.005) (Table 2, model 1). The association was attenuated after further multivariate adjustments for potential confounders but the trend towards higher risk remained borderline statistically significant (*P*-trend = 0.054) (Table 2, model 2). There was no evidence for non-linearity (Fig. 1). Further adjustment for serum albumin as a potential marker for poor nutritional status had no impact on the associations (data not shown).

**Table 2:** Risk of an incident infection according to the quartiles of serum Cu/Zn-ratio and Cu and Zn concentration

Serum parameter	Quartile of serum parameter				*P*-trend
	1	2	3	4	
Serum Cu/Zn-ratio	0.59–1.03	1.04–1.17	1.18–1.33	1.34–2.97	
N of events/subjects	140/494 (28.3%)	157/493 (31.8%)	174/495 (35.2%)	165/493 (33.5%)	
IR/1000 PY	13.8	16.0	18.4	19.0	
Model 1	1	1.14 (0.91–1.43)^a^	1.33 (1.06–1.66)	1.35 (1.07–1.69)	0.005
Model 2	1	1.08 (0.86–1.36)	1.28 (1.02–1.60)	1.21 (0.96–1.53)	0.054
Serum Cu (μmol/L)	7.24–15.58	15.59–17.31	17.32–18.88	18.89–36.51	
N of events/subjects	124/453 (27.4%)	182/573 (31.8%)	156/475 (32.8%)	174/474 (36.7%)	
IR/1000 PY	13.2	15.9	17.6	20.7	
Model 1	1	1.17 (0.93–1.48)	1.30 (1.03–1.65)	1.60 (1.27–2.02)	< 0.001
Model 2	1	1.13 (0.90–1.43)	1.20 (0.94–1.52)	1.39 (1.10–1.75)	0.005
Serum Zn (μmol/L)	8.26–13.15	13.16–14.22	14.23–15.30	15.31–24.78	
N of events/subjects	177/493 (35.9%)	141/499 (28.3%)	161/493 (32.7%)	157/490 (32.0%)	
IR/1000 PY	20.2	14.4	16.3	16.2	
Model 1	1	0.68 (0.54–0.85)	0.80 (0.65–1.00)	0.85 (0.69–1.06)	0.307
Model 2	1	0.71 (0.57–0.89)	0.84 (0.67–1.04)	0.83 (0.67–1.04)	0.218

^a^Values are hazard ratio (95% confidence interval)

*IR* incidence rate, *PY* person-years

Model 1: adjusted for age and examination year

Model 2: adjusted for model 1 and history of coronary heart disease, stroke, cancer or diabetes (yes/no); smoking (never smoker, previous smoker, current smoker < 20 cigarettes/day, current smoker ≥ 20 cigarettes/day); education (years); income (euros/year); intake of alcohol (g/week); leisure-time physical activity (kcal/day); and body mass index

In the analysis with serum Cu and Zn concentrations separately, higher serum Cu concentration was associated with higher risk of an incident infection. In the multivariable-adjusted model (Table 2, model 2), those in the highest versus the lowest serum Cu quartile had 39% higher risk of an incident infection (95% CI = 10–75%; *P*-trend = 0.005), without evidence for non-linearity (Fig. 1). No statistically significant associations were observed with serum Zn concentration (Table 2, Fig. 1).

In the sensitivity analysis, we evaluated the associations of Cu/Zn-ratio and serum Cu and Zn concentrations with the incidence of an infection during the first 10 years of follow-up, because the associations with a single measurement at baseline may be attenuated with a long follow-up period. After adjustment for potential confounders (Model 2), men in the highest versus the lowest Cu/Zn-ratio had 43% higher risk of an incident infection (202 events) (95% CI = 0.95–116%; *P*-trend = 0.043) (Supplementary table). Those in the highest versus the lowest serum Cu quartile had 50% higher risk (95% CI = 0.98–130%; *P*-trend = 0.062) (Model 2, Supplementary table). In these analyses also serum Zn concentration was associated with the risk of an incident infection, with 35% lower risk in the highest compared to the lowest quartile (95% CI = 4–55%). However, although the *P* value for trend across the quartiles was statistically significant (*P*-trend = 0.035), the lower risk was observed already in the second quartile and the risk did not decrease further with increasing serum Zn concentration (Supplementary table). Because the serum concentrations of both Cu and Zn are affected by the acute phase response, we also analysed the associations after excluding the infections that occurred during the first two years of follow-up (n = 37). This had only little impact on the associations: the HR (95% CI) in the highest versus the lowest quartile was 1.16 (0.94–1.50, *P*-trend = 0.06) for the Cu/Zn-ratio, 1.40 (1.10–1.78, *P*-trend = 0.005) for Cu and 0.89 (0.71–1.11, *P*-trend = 0.47) for Zn. If the analyses were restricted to the men without history of ischemic heart disease, stroke, cancer or diabetes (n = 1424, 434 incident infections), the association with Cu/Zn-ratio was attenuated (Model 2, extreme-quartile HR = 1.22, 95% CI = 0.91–1.63; *P*-trend = 0.24), but the association with Cu was stronger (extreme-quartile HR = 1.50, 95% CI = 1.14–1.99; *P*-trend = 0.01). There was no statistically significant association between serum Zn and incident infections in these analyses, either (extreme-quartile HR = 0.88, 95% CI = 0.67–1.16; *P*-trend = 0.57). Finally, we evaluated whether serum albumin concentration, BMI or smoking would modify the associations. The only statistically significant interaction was observed between BMI and serum Zn concentration. Serum Zn concentration was associated with a lower risk among men with BMI above the median (BMI > 26.4 kg/m^2^, Model 2 extreme-quartile HR = 0.70, 95% CI = 0.52–0.94; *P*-trend = 0.08), but no association was observed among the men with BMI below the median (extreme-quartile HR = 1.03, 95% CI 0.73–1.45; *P*-trend = 0.92) (*P*-interaction = 0.049). All other interactions were statistically non-significant (*P*-interactions > 0.15). Neither did we find evidence that the association of serum Cu with risk of an incident infection would be modified by serum Zn concentrations (*P*-interaction = 0.33) or vice versa (*P*-interaction = 0.49).

## Discussion

In this prospective, population-based cohort study, we found that a higher Cu/Zn-ratio and a higher concentration of serum Cu were associated with a higher risk of an incident infection requiring hospitalization, in middle-aged and older men from Eastern Finland. Serum Zn concentration was associated with the risk only in the analysis with a shorter follow-up of 10 years but not in the analyses with the full follow-up of an average 19 years. To our knowledge, the present study is the first to report on the relation between Cu/Zn-ratio and infection incidence in a population-based setting.

The observation of the higher risk with higher Cu/Zn-ratio may have various reasons. For the proper functioning of the innate and adapted immune response both of these micronutrients and their reciprocal relation are essential [25]. The optimum of the serum Cu/Zn-ratio lies between 0.7 and 1.0 [25], and also in our study the risk started to increase after the ratio exceeded 1.0 (Fig. 1). An increased serum Cu/Zn-ratio has been suggested as a valuable clinical marker in several case–control studies with bacterial, viral and parasitic infections [26–28], and in cohort studies with risk of all-cause, cancer, cardiovascular or HIV-1 mortality [11, 12, 15, 29]. Elderly are more susceptible to infections than younger people, and infections are an important cause not only for morbidity and mortality but also for hospital-admissions in elderly individuals [30]. Partly, this is due to the age-related weakening of the immune system (immunosenescence) [31]. Both Cu and Zn participate in antioxidant stress modulation [32, 33], and Cu/Zn-ratio had been used as a marker of oxidative stress burden [34]. A higher Cu/Zn-ratio may thus reflect an elevated oxidative stress burden, which may boost the age-related weakening of the immune system and contribute to the susceptibility to infections during ageing. Furthermore, it has been suggested that the increase of the serum Cu/Zn-ratio seems to be a frequent phenomenon related to age, chronic diseases and their pathological changes [9].

It has been suggested that the serum Cu/Zn-ratio is a superior marker of events to either serum Cu or Zn concentration separately [35]. However, in the present study serum Cu concentration had a stronger association with incidence of an infection than the serum Cu/Zn-ratio. The mean Cu concentration of the subjects in this study (17.4 μmol/L) corresponds well to the mean Cu concentrations for men obtained in studies in several European countries [36]. Of the subjects, 4.5% have higher Cu concentration than the upper reference value of Cu (22.0 μmol/L). The concentration of serum Cu is typically elevated in inflammation and infections [8], but the specific reasons for this phenomenon are still not clarified in detail [29]. The high concentration of serum Cu corresponds to the elevation of ceruloplasmin in serum. Ceruloplasmin is elevated in acute phase response [37]. As ceruloplasmin plays an important role in iron metabolism, it has been proposed that the high serum concentration of ceruloplasmin could rather reflect its role in iron mobilization and homeostasis and be the consequence of iron sequestration from microbes [38]. One potential explanation for the association between serum Cu concentration and infection incidence could thus be reverse causality. However, this may not be a likely explanation, because excluding the incident infections that occurred during the first 2 years of follow-up did not affect the associations with Cu.

The serum Zn concentration is typically decreased in infections [39]. This may be due to Zn deficiency [6], defined as a serum Zn concentration below 10.7 μmol/L [40], or due to the nutritional immunity [41], as serum Zn is redistributed through activation of inflammatory cytokines in particular into the liver [7, 42]. Furthermore, a decrease of serum Zn concentration and consequently an increase of Cu/Zn-ratio have been observed with advancing age [17]. It has been claimed that free-living elderly are prone to mild Zn deficiency [5, 18]. This may be linked to several factors associated with ageing, such as insufficient dietary Zn intake (e.g. problems of mastication leading to avoidance of Zn-rich foods like red meat), reduced intestinal absorption or increased losses (diarrhea, diuretics) [18]. However, in our study there were no major differences in dietary intakes across quartiles of serum Cu/Zn-ratio, including Zn intake (Table 1), suggesting that poor nutritional status is not a likely explanation for our findings. Neither did adjustment for serum albumin as a possible marker for poor nutritional status have any impact on the risk of an incident infection. Also a mild Zn deficiency has an adverse influence on the immune system. Similar alterations in immune system seen in Zn deficiency and immunosenescence suggest that these might be related phenomena [5, 18]. Additionally, a chronic exposure to cadmium can lead to a reduced renal reabsorption of Zn and result in decreased serum Zn concentration and increased Cu/Zn-ratio notably in smokers [19]. However, in our analyses Zn concentration was not associated with higher risk of an infection among the smokers, either. In this study we found an inverse association between serum Zn concentrations and risk of an infection with the shorter, 10-year follow-up, although without evidence for dose–response. This might be due to the attenuation of the relation during the longer follow-up time. Another potential explanation could be the small proportion of subjects (1.7%), which had serum Zn concentration lower than 10.7 μmol/L defined as Zn deficiency [40] and that higher Zn concentrations seem not to lower the risk of incident infections. The amount of Zn deficient subjects is less than the proportions reported in other studies varying from 3 to 4.8% in free-living elderly populations in Europe [40]. In the current work the mean serum Zn concentration (14.4 μmol/L) is in the same range as Zn values for men reported in other studies, such as in the ZENITH study [40], NHANES II Study (1976–1980) [43], and AREDS study [44]. There is no clear explanation for our finding that higher serum Zn concentration was associated with a lower risk among men with higher BMI, but because of the many interaction analyses, this could also be a chance finding.

The strengths of the current study are the long follow-up with a large number and detailed information of incident infections, population-based recruitment, and extensive examinations for potential confounders. The potential weakness is the single measurement of serum Cu and Zn concentrations at baseline, which may attenuate the associations during a long follow-up. This is supported by the stronger associations in the analyses with a shorter follow-up. As the study population included only middle-aged and older men from Eastern Finland, generalization to other populations should be done with caution. This includes potential gender-specific differences. In the present study the most common infection was pneumonia. It has been observed that the incidence of pneumonia in Europe is higher in men than in women [45]. In general, urinary tract infections (UTI) are the most common infections of the elderly [46]. Taking into account that the prevalence ratio of UTI between older women and men is 2:1 [47], it is conceivable that the incidence of UTI would be higher if women were included in the study population. Furthermore, it has been shown that gender differences exist in metal blood concentrations: Cu concentration is significantly higher in elderly women, while Zn concentration is higher in elderly men [48].

In conclusion, our data suggest that a higher serum Cu/Zn-ratio is associated with increased risk of incident infection in middle-aged and older men. However, because serum Cu concentration had a stronger association with increased risk, this suggests that serum Cu concentration alone could be a better marker for future risk of an infection. Further research in other study populations is required to verify the results.

## Electronic supplementary material

Below is the link to the electronic supplementary material.

Supplementary material 1 (DOCX 18 kb)
