# Supplementary material for: Serum copper-to-zinc-ratio and risk of incident infection in men: the Kuopio Ischaemic Heart Disease Risk Factor Study
Source: Eur J Epidemiol. 2020 May 13;35(12):1149–56. doi: 10.1007/s10654-020-00644-1 (PMC7762744; doi:10.1007/s10654-020-00644-1)
Supplement: Supplementary file 1 — Supplementary material 1 (DOCX 18 kb) [file 10654_2020_644_MOESM1_ESM.docx]

**Supplementary table.** Serum Cu/Zn-ratio, serum Cu and Zn concentrations and risk of an incident infection in 1975 men of the KIHD during the average follow-up of 10 years.

|  | Quartile of serum parameter | | | |  |
| --- | --- | --- | --- | --- | --- |
| Serum parameter | 1 | 2 | 3 | 4 | *P*-trend |
| **Serum Cu/Zn-ratio** | 0.59–1.03 | 1.04–1.17 | 1.18–1.33 | 1.34–2.97 |  |
| N of events/subjects | 39/494 (7.9%) | 42/493 (8.5%) | 58/495 (11.7%) | 63/493 (12.8%) |  |
| IR/1000 PY | 7.7 | 8.4 | 11.8 | 13.5 |  |
| Model 1 | 1 | 1.07 (0.69-1.66)^a^ | 1.49 (0.99-2.23) | 1.64 (1.10-2.45) | 0.005 |
| Model 2 | 1 | 1.06 (0.69-1.64) | 1.43 (0.95-2.15) | 1.43 (0.95-2.16) | 0.043 |
|  |  |  |  |  |  |
| **Serum Cu** (μmol/L) | 7.24–15.58 | 15.59–17.31 | 17.32–18.88 | 18.89–36.51 |  |
| N of events/subjects | 33/453 (7.3%) | 55/573 (9.6%) | 51/475 (10.7%) | 63/474 (13.3%) |  |
| IR/1000 PY | 7.4 | 9.4 | 10.8 | 13.8 |  |
| Model 1 | 1 | 1.27 (0.82-1.96) | 1.44 (0.93-2.24) | 1.84 (1.20-2.80) | 0.003 |
| Model 2 | 1 | 1.22 (0.79-1.89) | 1.29 (0.83-2.02) | 1.50 (0.98-2.30) | 0.062 |
|  |  |  |  |  |  |
| **Serum Zn** (μmol/L) | 8.26–13.15 | 13.16–14.22 | 14.23–15.30 | 15.31–24.78 |  |
| N of events/subjects | 72/493 (14.6%) | 39/499 (7.8%) | 44/493 (8.9%) | 47/490 (9.6%) |  |
| IR/1000 PY | 15.4 | 7.8 | 8.8 | 9.5 |  |
| Model 1 | 1 | 0.51 (0.34-0.75) | 0.59 (0.41-0.86) | 0.66 (0.45-0.95) | 0.030 |
| Model 2 | 1 | 0.55 (0.37-0.81) | 0.64 (0.44-0.94) | 0.65 (0.45-0.96) | 0.035 |

^a^Values are hazard ratio (95% confidence interval).

IR, incidence rate; PY, person-years

Model 1: adjusted for age and examination year.

Model 2: adjusted for model 1 and history of coronary heart disease, stroke, cancer or diabetes (yes/no); smoking (never smoker, previous smoker, current smoker <20 cigarettes/day, current smoker >20 cigarettes/day); education (years); income (euros/year); intake of alcohol (g/week); leisure-time physical activity (kcal/day); and body mass index.
